# Primary progressive aphasia: six questions in search of an answer

**DOI:** 10.1007/s00415-023-12030-4

**Published:** 2023-10-31

**Authors:** Christopher R. S. Belder, Charles R. Marshall, Jessica Jiang, Salvatore Mazzeo, Anthipa Chokesuwattanaskul, Jonathan D. Rohrer, Anna Volkmer, Chris J. D. Hardy, Jason D. Warren

**Affiliations:** 1 Dementia Research Centre, Department of Neurodegenerative Disease, UCL Queen Square Institute of Neurology, University College London, 8 – 11 Queen Square, London, WC1N 3BG UK; 2 UK Dementia Research Institute at UCL, UCL Queen Square Institute of Neurology, University College London, London, UK; 3 Adelaide Medical School, The University of Adelaide, Adelaide, South Australia Australia; 4 Preventive Neurology Unit, Wolfson Institute of Population Health, Queen Mary University of London, London, UK; 5 Department of Neuroscience, Psychology, Drug Research and Child Health, University of Florence, Azienda Ospedaliera-Universitaria Careggi, Florence, Italy; 6 Division of Neurology, Department of Internal Medicine, King Chulalongkorn Memorial Hospital, Thai Red Cross Society, Bangkok, Thailand; 7 Cognitive Clinical and Computational Neuroscience Research Unit, Faculty of Medicine, Chulalongkorn University, Bangkok, Thailand

**Keywords:** Primary progressive aphasia, Semantic dementia, Logopenic aphasia, Frontotemporal dementia, Alzheimer’s disease

## Abstract

Here, we review recent progress in the diagnosis and management of primary progressive aphasia—the language-led dementias. We pose six key unanswered questions that challenge current assumptions and highlight the unresolved difficulties that surround these diseases. How many syndromes of primary progressive aphasia are there—and is syndromic diagnosis even useful? Are these truly ‘language-led’ dementias? How can we diagnose (and track) primary progressive aphasia better? Can brain pathology be predicted in these diseases? What is their core pathophysiology? In addition, how can primary progressive aphasia best be treated? We propose that pathophysiological mechanisms linking proteinopathies to phenotypes may help resolve the clinical complexity of primary progressive aphasia, and may suggest novel diagnostic tools and markers and guide the deployment of effective therapies.

**Supplementary Information:**

The online version contains supplementary material available at 10.1007/s00415-023-12030-4.

## Introduction

In the 40 years since their modern rediscovery [1], the primary progressive aphasias (PPA) or ‘language-led dementias’ have transformed our picture of aphasia and selective neural system vulnerability to degenerative proteinopathies. In 2018 [2], we presented a general overview and clinical approach to PPA and its variant syndromes in this journal. Despite considerable ongoing clinical and research attention, they are still, in many ways, mysterious disorders. Though uncommon (with a collective population prevalence conservatively estimated at around three cases per 100,000 [3, 4]), they are immensely disabling and distressing, tending to affect people in later middle life and wreaking havoc on social and occupational functioning. These diseases of communication failure present unsolved challenges for neurobiological characterisation, diagnosis and management, liable to misinterpretation and delayed recognition [5, 6].

Here, we present an update on PPA, intended as a companion to our earlier paper [2] and directed to practising clinicians who see and care for these patients. We pose a series of ‘six questions in search of an answer’: each represents a key problem that has long bedevilled clinical practice in PPA, continues to provoke controversy and is likely to shape future progress. For each question (we argue), recent developments challenge conventional assumptions about these diseases. We conclude with a prospect of future progress.

## How many PPA syndromes are there—and is a syndromic diagnosis even useful?

### Core syndromes

**Fig. 1:**
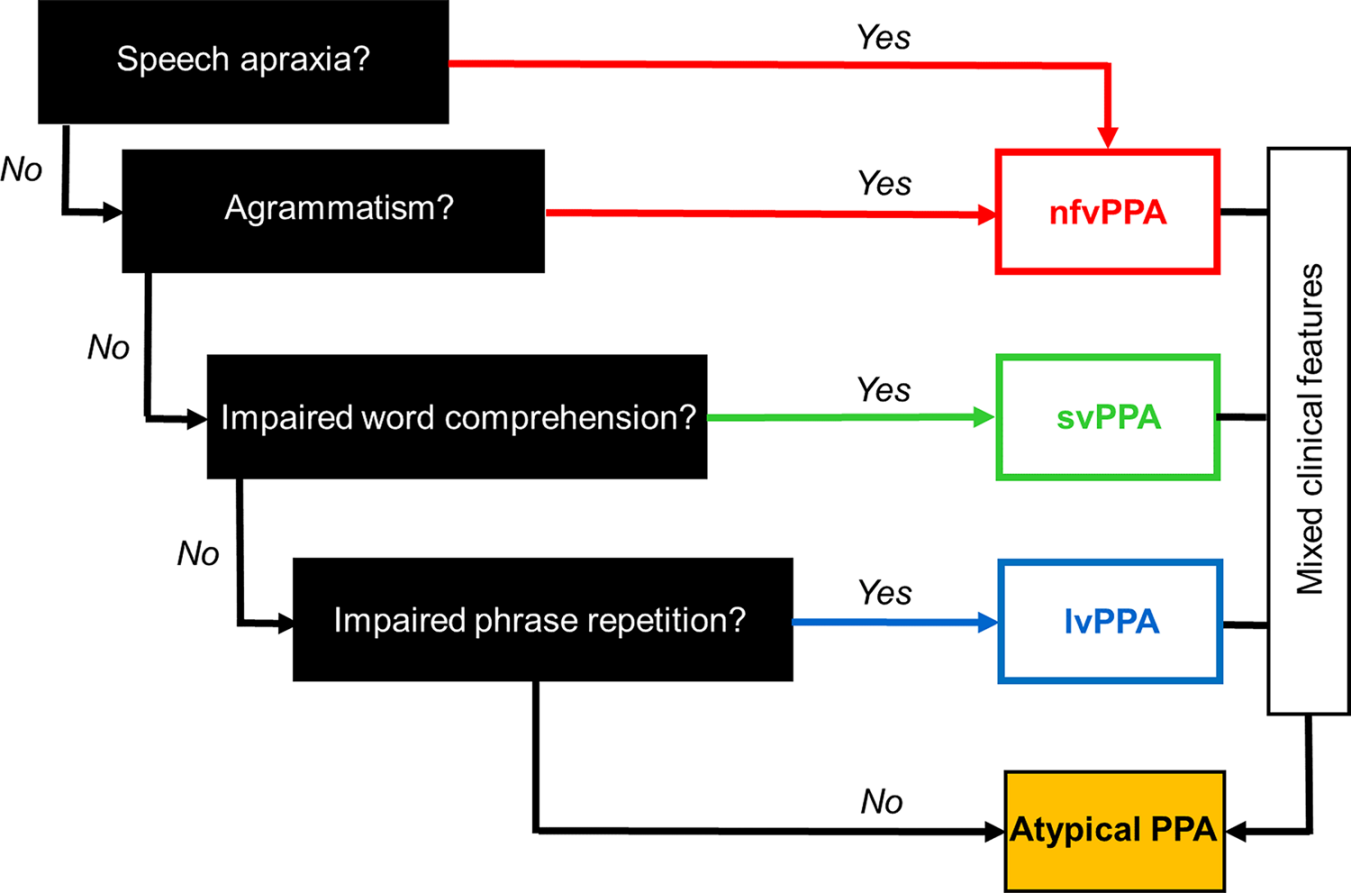
A ‘roadmap’ for making a syndromic diagnosis of primary progressive aphasia (PPA) at the bedside, in patients presenting with progressive speech and/or language impairment as leading and dominant symptoms. On the left of the Figure, we list key clinical features that are most discriminating for major variant syndromes of primary progressive aphasia (see also Table 1), according to current consensus diagnostic criteria [7] (see Table S1). Speech production impairment (apraxia and/or grammatical errors in speaking or writing) points to the nonfluent/agrammatic variant (nfvPPA), impaired single-word comprehension to the semantic variant (svPPA), and impaired repetition of phrases (disproportionate to single words) to the logopenic variant (lvPPA). Speech apraxia, agrammatism and word comprehension impairment are elicited on history and examination; impaired phrasal repetition must be confirmed on examination. Note that clinical features often seen in PPA but less useful in differentiating syndromes have not been included here (e.g. anomia is prominent in both svPPA and lvPPA). Cases may not conform to a single canonical syndrome (Atypical PPA); this may be due to relatively circumscribed language impairments that lack additional features (e.g. dynamic aphasia and progressive pure anomia), more complex mixed language phenotypes, or the presence of prominent non-language features (see text). Further investigations are indicated following the clinical syndromic diagnosis (see Fig. 2), to substantiate the bedside impression and fully characterise the syndrome (neuropsychometry where available; brain MRI in all cases), and to identify the underlying proteinopathy with a view to symptomatic treatment (Alzheimer’s disease biomarkers) or genetic counselling (where clinically appropriate). Adapted under a CC-BY 4.0 license from: Marshall et al., *J Neurol* 2018; 265: 1474–1490

PPA remains a quintessentially clinical diagnosis. Three canonical, clinico-anatomical variant syndromes of PPA are codified in the 2011 international consensus criteria developed by Gorno-Tempini et al. [7] (summarised for reference in Table S1). The nonfluent/agrammatic variant (nfvPPA) is led by impaired speech production with articulatory and/or grammatical errors, characteristically associated with predominantly left-sided anterior peri-Sylvian atrophy. The semantic variant (svPPA) is led by loss of vocabulary and impaired word knowledge due to a broader problem with semantic memory, consistently associated with focal, predominantly left-sided anterior, mesial and inferior temporal lobe atrophy. The logopenic variant (lvPPA) is led by word finding pauses, anomia and impaired verbal (phonological) working memory, manifesting as disproportionate difficulty repeating phrases over single words; its key neuroanatomical locus is the left temporo-parietal junction, though the extent and asymmetry of atrophy here varies widely between patients. A practical implementation of the diagnostic criteria is diagrammed as a ‘roadmap’ for rapid bedside diagnosis of PPA in Fig. 1.

Although the consensus diagnostic criteria have largely shaped current clinical and research practice in PPA, both clinical experience and published case series [8–12] indicate that there are frequent and significant exceptions to the standard formulation, variously designated ‘atypical PPA’, ‘mixed PPA’, ‘PPA-unclassifiable’, ‘PPA-extended’ or ‘PPA not otherwise specified’. In up to around a third of cases, the patient’s presentation may not fulfil criteria for a single canonical syndrome (due to fragmentary or mixed features) or prominent non-linguistic symptoms may accompany the language disturbance. Any consideration of the ‘atypicality’ of PPA should acknowledge the dynamic nature of these diseases: the duration of symptoms is important, as the language deficits of PPA syndromes tend to converge over time [13]. However, a mixed PPA phenotype may be evident from the earliest stages of the illness [11, 12]; even otherwise ‘typical’ nfvPPA, for example, can be accompanied by impaired single-word comprehension [14, 15], and lvPPA by agrammatism [16]. Among patients fulfilling criteria for a given canonical syndrome, there is substantial individual variation in the tempo, ordering and salience of particular features, and this heterogeneity is particularly marked for nfvPPA [5].

Responses to this problem reflect broadly dichotomous ‘splitter—lumper’ perspectives.

### Syndrome splitting

From a ‘splitter’ perspective, the existence of separable sub-syndromes within the canonical syndromic variants of PPA has been argued for some time. Sub-syndromes are best defined for nfvPPA, splitting the cardinal speech apraxic and agrammatic components of the canonical syndrome. Primary progressive apraxia of speech (PPAOS) [15, 17–19] is characterised by reduced articulatory agility, variable phonemic distortions and ‘groping’ towards the target sound initially without associated linguistic deficits; there is often accompanying apraxia of orofacial movements (such as yawning or whistling to command) disproportionate to any limb apraxia [20]. Further subdivision of PPAOS into phonetic, prosodic and mixed variants has been proposed [21], while other motor speech disturbances (including various forms of dysarthria and stuttering) have been reported in nfvPPA and atypical PPA [22]; however, such fine-grained differentiation is difficult for most clinicians (and of uncertain clinical value). The second, major sub-syndrome of nfvPPA, progressive agrammatic aphasia without apraxia of speech, is less common than PPAOS [23, 24]: both in this variant and PPAOS, other features of nfvPPA tend to manifest later in the illness, though not invariably. Rarer sub-syndromes are also seen (see [2]). While verbal adynamia—impaired generation of propositional language—often accompanies nfvPPA (particularly in the context of parkinsonism [25, 26]), it can present in pure form years before the development of other aphasic or neurological deficits: ‘primary progressive dynamic aphasia’ [27, 28].

Sub-syndromes of lvPPA have also been delineated [15, 29, 30] though less well defined clinically than in nfvPPA: these vary in the severity of anomia, single-word repetition, word comprehension and expressive agrammatism. There is no sharp clinical demarcation between lvPPA, typical memory-led Alzheimer’s disease and its major ‘visual’ variant, posterior cortical atrophy [31–33]. Non-linguistic cognitive problems—including difficulties with episodic memory (forgetfulness, repetitiveness), praxis (e.g. use of household gadgets) and visuospatial awareness (e.g. locating items by sight, finding exits)—are more common at an earlier stage than in other PPA syndromes [12, 31, 34–36], and with disease evolution, most patients will meet criteria for Alzheimer’s dementia [12].

By contrast, svPPA is a highly coherent and distinctive syndrome; however, its demarcation from the closely related syndrome of right temporal lobe atrophy is an active issue [37–41]. Patients presenting with a right-sided atrophy profile mirroring that seen in typical svPPA usually have a clinical picture dominated by socio-emotional behavioural dysfunction, often accompanied by cognitive deficits (such as progressive prosopagnosia) that overlap with those in svPPA and may be at least partly semantically grounded [38, 42]. Indeed, similar behavioural and nonverbal cognitive deficits develop in svPPA as the disease advances; the right anterior temporal lobe is involved early in svPPA [43] and increasingly prominently as the illness progresses [37, 44], suggesting that the ‘left’ and ‘right’ temporal lobe atrophy syndromes lie on the same clinico-anatomical spectrum. On the other hand, there may be phenotypic features uniquely associated with the preferential targeting of the right anterior temporal lobe, linked to impaired homeostatic and hedonic regulation [39].

### Beyond syndromes?

A nuanced version of the ‘lumper’ perspective has recently gained ascendancy in the nosology of PPA. The application of multidimensional methods such as principal component analysis to neuropsychological datasets derived from large PPA cohorts has highlighted the extent to which the canonical syndromes overlap and the graded nature of deficits within as well as between diagnostic groups [45]. Whereas (in line with clinical intuition) svPPA forms a distinct diagnostic category (based on within-group homogeneity and strong between-group differences), the categories are less clear for the other syndromes. This approach emphasises trans-syndromic factors that map onto particular cognitive factors (e.g. motor speech, phonological and semantic) over the canonical syndromes enshrined in the Gorno-Tempini criteria. lvPPA has been particularly foregrounded as a multidimensional disorder with highly heterogeneous individual profiles of cognitive impairment, reflecting the conjunction of differentiated linguistic and non-linguistic processes [31, 46]. One important practical message emerging from this work is that non-linguistic markers may assist in the clinical diagnosis of PPA (for example, nonverbal memory impairment discriminates lvPPA from nfvPPA [47]). As clinicians, we see considerable value in syndromic diagnosis (sensitively conveyed) for patients and caregivers. There is no doubt, however, that the diagnosis of PPA, and understanding what that means for the affected individual, entails much more than the application of a label.

## Are these truly ‘language-led’ dementias?

### Non-linguistic deficits

From a cognitive perspective, the essentially ‘aphasic’ status of each of the canonical PPA syndromes might be challenged. In the case of lvPPA, the appearance of closely linked non-aphasic cognitive deficits very early in the illness may warrant a reformulation of this syndrome to incorporate domain-general and domain-selective cognitive processes beyond language [31]. svPPA might be better described by its older name, ‘semantic dementia’ for this is integrally a disorder of semantic memory [48–50]—the dedicated memory system that mediates our knowledge of words, objects and concepts. Patients with svPPA in our experience invariably lose understanding of nonverbal signals, visual and other sensory objects as well as social concepts and ‘rules’, as the illness evolves—in keeping with a ‘pan-modal’ semantic impairment [51–55]. This syndrome might manifest as a disorder of language because, in general, language makes the most taxing demands on semantic processing in everyday life, or perhaps because a problem with language is relatively easily recognised and characterised. Even within the nfvPPA spectrum, the core deficit in the major variant—PPAOS—may lie principally with motor speech execution rather than with language processing per se [56]; written or typed communication is often initially largely intact, as indeed is verbal ideation [57], though in our experience aphasic deficits do supervene later.

It is well recognised that non-linguistic features commonly develop later in the course of PPA [12, 58]. Many patients with nfvPPA (or PPAOS) will develop atypical parkinsonism with clinical features of progressive supranuclear palsy or corticobasal syndrome [12, 18, 59, 60]. Features of motor neuron disease develop much less frequently, but have been variably reported to show a stronger association with nfvPPA [61] or svPPA [62]. Behavioural changes overlapping those seen in the behavioural variant of frontotemporal dementia (such as disinhibition, apathy and altered eating behaviour) are common across subtypes, most strikingly in svPPA [2, 12, 63]. Increasingly, however, nonverbal accompaniments are recognised even in early stage PPA which nevertheless fulfils criteria for a canonical syndrome [7]. It is of course understandable that the onset of speech and language difficulties should prompt the patient or their family to seek medical attention; however, this leaves open the possibility that less well defined (or less readily acknowledged) symptoms might actually lead the syndrome, or at least evolve in tandem with language dysfunction. Certain neuropsychiatric symptoms—such as compulsions in svPPA, depression in nfvPPA and anxiety in lvPPA—are exhibited by a substantial proportion of patients even at presentation [6, 12]. Moreover, early behavioural changes may be more subtle, yet still significant for daily life function (for example, an impoverished sense of humour in svPPA; withdrawal from social activities in lvPPA [5, 64]).

### Hearing changes

Hearing impairment has emerged as an important non-linguistic issue in PPA, in line with the known close relations between language and auditory brain function [65]. Difficulty hearing in noisy environments is reported early in the illness across PPA syndromes, and all show reduced comprehension of acoustically degraded speech compared with healthy older listeners; these difficulties are most marked in lvPPA and nfvPPA [5, 66, 67]. Abnormal ‘auditory hedonic’ behaviours (strong liking or aversion for music or other sounds) frequently develop in svPPA [68]. Additional, relatively specific auditory cognitive phenotypes have been linked to each of the canonical syndromes. Patients with nfvPPA have reduced detection of sounds [69] and impaired perception of fundamental sound properties such as pitch, timbre and rhythm [53, 70–72] and their elaboration in prosody and music [73–75]. svPPA is associated with environmental sound agnosia [53] and phonagnosia (impaired voice recognition) [76], as well as tinnitus and hyperacusis [77]. lvPPA is particularly associated with impaired perception of phonemes [71, 78], while performance on hearing tasks more generally is modulated by auditory working memory capacity [70]. Atypical PPA presentations with prominent auditory impairments have also been identified, including progressive phonagnosia [79, 80], progressive word deafness (disproportionately impaired comprehension of spoken versus written words) [1, 81–83], and generalised auditory agnosia [84]. Taken together, these diverse non-linguistic auditory deficits suggest that PPA syndromes might be best characterised as pervasive ‘communication’ disorders: language output deficits in all three major PPA syndromes are likely to be influenced by disordered complex sound processing. The linkage between auditory and language output deficits is still poorly understood—here, speech repetition paradigms developed for lvPPA might perhaps serve as a model.

## How can we diagnose (and track) PPA better?

### Making the diagnosis

**Fig. 2:**
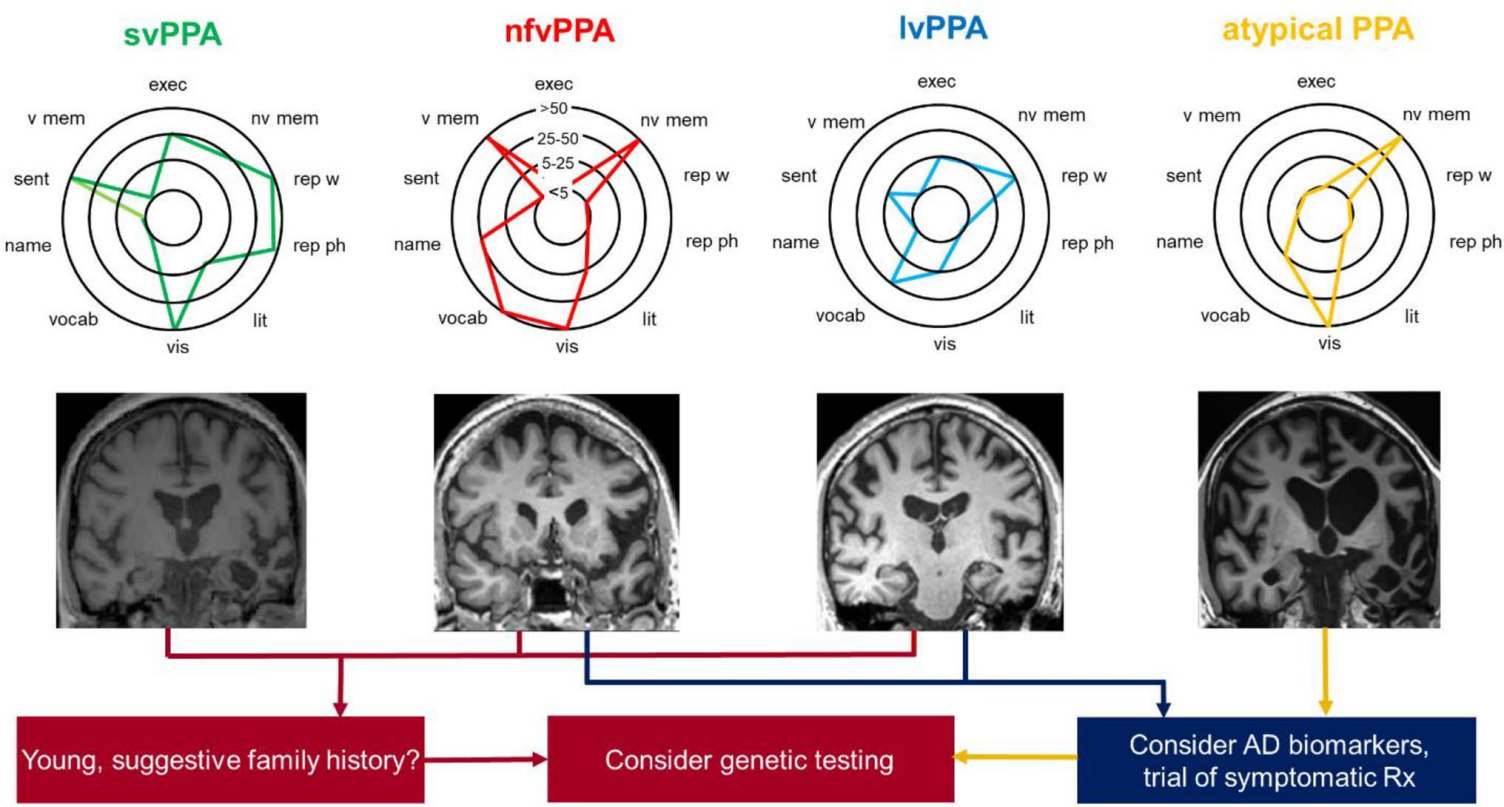
Next steps after bedside diagnosis in syndromes of primary progressive aphasia: semantic variant primary progressive aphasia (svPPA), nonfluent/agrammatic variant (nfv)PPA, logopenic variant (lv)PPA and atypical or ‘mixed’ PPA. Where available, assessment by a neuropsychologist is very valuable in fully defining and quantifying the cognitive phenotype, over linguistic as well as non-linguistic domains. The ‘target diagrams’ (top panels) show typical profiles of neuropsychological test performance for each syndrome; concentric circles indicate percentile scores relative to a healthy age-matched population and distance along the radial dimension represents level of functioning in the cognitive domains assessed (**exec**, executive skills; **lit,** literacy skills (spelling, arithmetic); **name**, naming; **nv mem**, nonverbal memory; **rep ph**, repetition of phrases; **rep w**, repetition of single words; **sent**, sentence processing (construction and comprehension); **vis**, visuo-spatial; **v mem**, verbal memory; **vocab**, vocabulary (single-word comprehension)). Brain imaging (wherever possible, MRI) is an essential part of the diagnostic workup of any patient with suspected PPA; coronal T1-weighted brain MRI sections representing characteristic atrophy profiles in each syndrome are shown (middle panels; left hemisphere presented on the right). In svPPA, the profile of asymmetric (predominantly left-sided) anterior, mesial and inferior temporal lobe atrophy is highly consistent, whereas atrophy profiles in nfvPPA (predominantly left inferior frontal, insular and anterior superior temporal gyrus atrophy) and lvPPA (predominantly involving left posterior superior temporal and inferior parietal cortices) are much more variable between individual patients. In patients with lvPPA, mixed PPA and nfvPPA, we have a low threshold for trialling a symptomatic therapy for Alzheimer’s disease (AD), such as donepezil. In younger patients, assessing AD biomarkers in CSF or brain amyloid PET is likely to provide diagnostically relevant information, and genetic testing for a mutation in the frontotemporal dementia spectrum is also a consideration (see text), particularly where there is a suggestive family history, atypical clinical features or a strikingly asymmetric atrophy profile (as in the patient with mixed PPA here, who had a pathogenic progranulin gene mutation). The possibility of conjoint pathologies should be kept in mind [30]. Adapted under a CC-BY 4.0 license from: Marshall et al., *J Neurol* 2018; 265: 1474–1490

Timely diagnosis of PPA resolves uncertainty for patients and families, enables future planning and unlocks access to services and support. The dawning of the age of disease-modifying therapies for neurodegenerative disease [85], with its imperative to diagnose dementias earlier and more accurately, has only amplified this issue. However, the clinical diagnosis of PPA is often challenging even for those with extensive experience of the syndromes. We present some clinical, cognitive and neuroimaging ‘fingerprints’ that we have found particularly useful in the early detection of PPA syndromes in Table 1, an approach to bedside syndromic diagnosis in Fig. 1 and an outline of ancillary investigations in Fig. 2.

**Table 1:** Some ‘fingerprints’ of primary progressive aphasia at diagnosis

Initial suspicion^a^	Is it primary progressive aphasia?		
Speech/language symptoms	Spelling errors (recent onset), work/social activities impacted, progressive decline		
Other symptoms	Difficulty using/learning devices, increased difficulty hearing in busy environments, changes in personality and inter-personal interactions (often subtle)^b^		

Syndromic diagnosis	nfvPPA	svPPA	lvPPA
Speech/language: history	Hesitant speech with errors of pronunciation and/or grammar, binary (especially ‘yes/no’) confusions	Asking the meaning of familiar words, keeping personal ‘dictionaries’	Losing thread of sentences, frequent tip-of-tongue hesitations in conversation, occasional jargon (neologisms)
Speech/language: examination	Speech apraxia: obviously effortful, distorted speech sounds with ‘groping’ after target sound, reduced articulatory agility (difficulty repeating syllable strings, e.g. ‘pa-ke-ta’) Grammatical errors^c^	Impaired single word comprehension^d^: unable to define words/identify items nominated by examiner Regularisation errors on reading aloud, e.g. sounding yacht as ‘yached’ (‘surface dyslexia’)	Impaired verbal working memory: errors repeating phrases/sentences, more prominent with increasing target length, despite intact repetition of single words; reduced digit span
Other clinical features^e^	Orofacial apraxia Neurological (especially extrapyramidal) signs^f^	Visual agnosia^g^ New interests (e.g. puzzles, numbers, music), clockwatching	Difficulty with route-finding Limb apraxia Nonverbal episodic memory impairment Visuospatial difficulties
Brain MRI atrophy signatures	Left anterior peri-Sylvian	Predominantly left anterior, mesial and inferior temporal^h^	Left posterior temporal/parietal

**Fig. 3:**
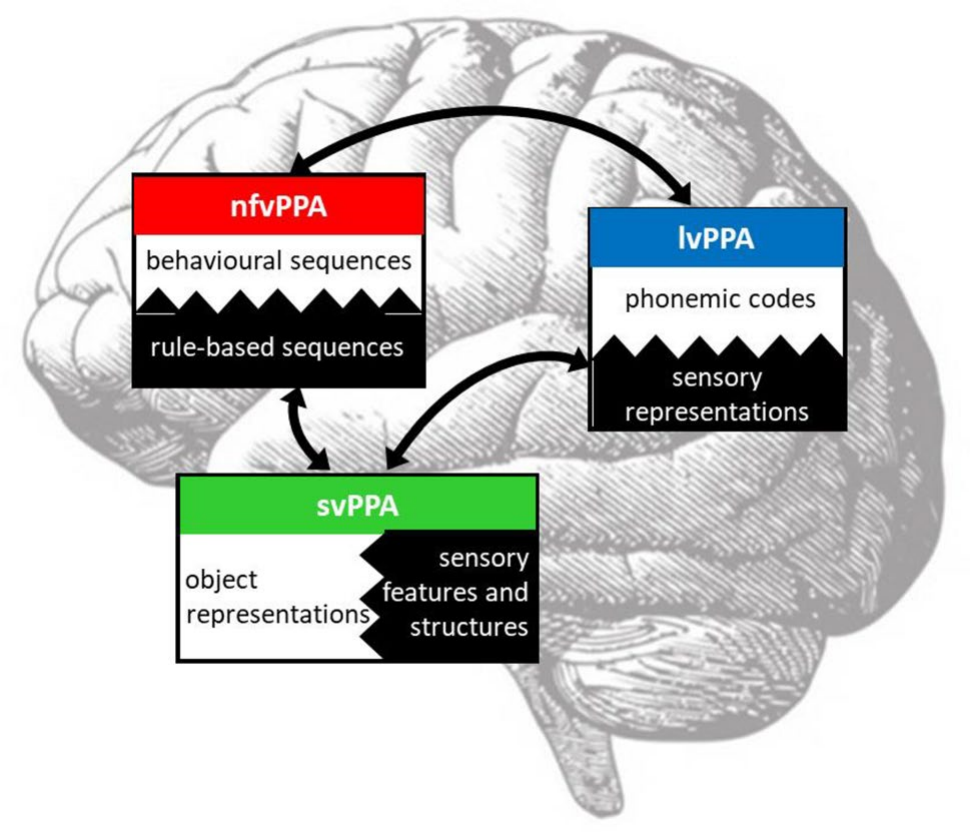
Proposed pathophysiology of primary progressive aphasia. The figure diagrams a lateral view of the left cerebral hemisphere, overlaid with core neural mechanisms that we propose underpin each of the major variant syndromes of primary progressive aphasia (PPA): nonfluent/agrammatic (nfvPPA), semantic (svPPA) and logopenic (lvPPA). Oblongs signify major processing ‘hubs’ within the language network: each instantiates a key neural ‘template-matching’ operation in which incoming data (represented by black hatching) is iteratively reconciled with stored predictions and transformed into an output (‘predictive coding’; see text). The bidirectional arrows represent the reciprocal exchange of data and predictions between core processing modules. Processing modules are organised hierarchically, in that representations of incoming sensory data in temporo-parietal junctional cortex (blue) are transformed into increasingly abstract conceptual representations in anterior temporal cortex (green) and may ultimately be used in generating a motor output via anterior peri-Sylvian mechanisms (red). The putative core physiological mechanism targeted in each syndrome gives rise to the essential features of the syndrome. In lvPPA, this mechanism is proposed to be the transformation of sensory data into phonological codes; if these codes are defective, they do not enter working memory normally and cannot be used to access other components of the language network (e.g. during naming). The semantic appraisal network, targeted in svPPA, stores multimodal representations corresponding to words, objects and concepts, based on the computation of higher-order regularities in sensory data: erosion of these semantic representations leads initially to loss of vocabulary and ultimately, a pan-modal impairment of semantic memory. In nfvPPA, the core pathophysiological mechanism may be impaired transformation of rule-based command sequences (such as those governing grammar and articulation) into behavioural routines (for example, because excessively rigid predictions lead to delayed error resolution [169]); Reproduced under a CC-BY 4.0 license from: Ruksenaite et al., *Curr Neurol Neurosci Rep* 2021; 21: 7

In this table, we summarise symptoms, signs and other features we have found useful in corroborating an initial clinical suspicion of primary progressive aphasia (PPA) and in differentiating between the major variant syndromes: nonfluent/agrammatic (nfvPPA), semantic (svPPA) and logopenic (lvPPA) (see also Figs. 1, 2, 3). The table is informed in part by surveys of caregivers for people living with primary progressive aphasia [5, 112]; however, not all patients with a given diagnosis will exhibit every feature, the chronology of deficits varies between individuals and nonverbal cognitive, behavioural and neurological features tend to become more prominent later in the illness (see text). **a** These features in our experience tend to develop early across syndromes and are useful in distinguishing PPA from other presentations with ‘word finding difficulty’, including health anxiety or functional cognitive disorder; **b** may include changes in humour, sociability, initiative, etc. as well as mood symptoms; **c** tends to be less prominent than speech apraxia but may sometimes lead the presentation, more reliably detected in written sentences if speech is very effortful; **d** this must be distinguished from anomia due to impaired word retrieval, which is very prominent in both svPPA and lvPPA and commonly seen across the PPA spectrum, and in other patients presenting to memory clinics; **e** by definition, initially subordinate to the language problem but of diagnostic and clinical relevance if present; **f** mild features of atypical parkinsonism (or less commonly, motor neuron disease) may be evident at presentation; **g** unable to recognise and/or demonstrate use of familiar items by sight); **h** we question the diagnosis of svPPA if the characteristic atrophy profile is absent, MRI features in other syndromes are substantially more variable

Early on, the key diagnostic problem is often to decide whether a ‘language-led dementia’ is present [86], and secondarily to make a syndromic diagnosis, as the major syndromes have their own management needs and implications for future care planning. The first symptoms of PPA are insidious, often subtle and tend to be dynamic (often brought out by particular situations, such as speaking before an audience or on the telephone). Some symptoms (such as ‘tip-of-the-tongue’ hesitations in lvPPA [87]) are commonly experienced by normal speakers. Some patients presenting with health anxiety or functional cognitive disorder describe prominent speech and language symptoms: aside from lack of progression (and sometimes, abrupt onset), positive clues to these diagnoses on history include elaborate symptom descriptions and discrepancies between the severity of the subjective complaint and daily life performance [88]. On examination, hallmarks of a functional speech disorder include stuttering or prosodic changes (‘childlike’ or a ‘foreign accent’) with pronounced variability (severe disruption interspersed with segments of normal speech) and distractibility [89]. The differential diagnosis of PPA is fairly limited. Canonical PPA variants do not have close equivalents in the classical syndromes of post-stroke aphasia (though detailed comparisons are surprisingly sparse [45, 90, 91]); this distinction is usually obvious on history, but may be confounded if a PPA syndrome is reported to have begun ‘suddenly’ (usually, this will be misattribution to a sentinel event) or where there are substantial comorbid cerebrovascular changes on brain MRI.

Clinical diagnostic ‘algorithms’ for PPA (see Figs. 1 and 2) tend to be directed towards established disease, though certain clues can be helpful in picking up problems at presentation (Table 1). Detailed and quantitative cognitive profiling by an expert neuropsychologist is invaluable in corroborating the initial clinical impression [92]; however, this is time-consuming and access to a neuropsychology department may be limited. Several cognitive instruments have been designed to facilitate the relatively rapid diagnosis of PPA syndromes in the clinic [93]. These include the Progressive Aphasia Language Scale (PALS) [94], the Sydney Language Battery [95], the Screening for Aphasia in NeuroDegeneration Battery (SAND) [96], the Progressive Aphasia RatIng Scale (PARIS) [97], and most recently, the Mini Linguistic State Examination [98] (which rests on an analysis of error types) and an online calculator based on the Addenbrooke’s Cognitive Examination version III [99] (which profiles subscores across language and other cognitive domains). These instruments have yet to be assessed head-to-head (or indeed, in combination) but we would emphasise that some clinical experience of PPA is required to use them optimally (interpretation of errors, for example, is often not straightforward). As always in neurology, the way a patient performs a test may be as informative as their score (the sense of effort associated with speaking, for example, is a distinguishing hallmark of nfvPPA (Table 1)). There is growing interest in automated diagnosis based on machine learning approaches [100] and the identification of digital markers that might detect very early stage PPA (based, for example, on daily life conversational language or device use [101, 102]), though their clinical utility has yet to be established.

### The value of neuroimaging

As with any dementia, brain imaging (wherever possible, MRI) is central to the diagnosis of PPA. In addition to excluding rare mimic syndromes, the profile of atrophy on MRI can substantiate the clinical impression (see Fig. 2). In our experience, typical svPPA is invariably associated with pronounced, selective (usually left-sided) anterior temporal lobe atrophy, even when the patient presents with only mild deficits. By contrast, in nfvPPA and lvPPA neuroanatomical correlation is less straightforward. Syndromic MRI signatures have been identified—in the nfvPPA spectrum, PPAOS and agrammatic aphasia have separable group-level atrophy profiles, predominantly involving premotor/supplementary motor cortex and inferior frontal/prefrontal cortices, respectively [17, 19, 23, 24]; while in lvPPA, involvement of left temporo-parietal cortex has been widely identified as a critical signature [31, 36, 103]. However, in individual patients MRI findings may be subtle or not in keeping with the level of clinical impairment. Serial MRI to detect progressive regional volume loss after an approximately 12-month interval may be helpful, as may metabolic neuroimaging with fluorodeoxyglucose positron emission tomography or single photon emission computed tomography: in nfvPPA and particularly in lvPPA, these techniques may detect regional neural hypometabolism ahead of any structural brain degeneration [104] but reporting is often not quantitative. Newer MRI techniques such as quantitative analysis of diffusion-weighted sequences to capture microstructural disorganisation show promise as sensitive markers of PPA [105] but have not yet entered clinical practice. Accurate interpretation of MRI and other neuroimaging modalities in PPA benefits from close acquaintance with this disease spectrum.

### Prognosis and staging

Related to the early diagnosis of PPA is the problem of prognosis, which remains very imprecise in the individual case. Both prognosis and care planning would be greatly facilitated by a clinical staging system for PPA [58]. However, this is a daunting proposition, due to the heterogeneous and dynamic, nonlinear trajectories of these diseases (patients and their caregivers often describe fluctuations in their ability to perform specific tasks from day to day and periods of relative stability or apparently more rapid decline) as well as the relatively loose mapping between clinical status and objective disease markers. Standard instruments for assessing dementia stage and severity rest primarily on concepts formulated by clinicians (rather than the lived experience of patients and caregivers) and do not assess language and communication functions in detail [106–110]. Clinical severity scales that are most relevant to PPA (such as the Progressive Aphasia Severity Scale (PASS) [111]) have limited coverage of non-linguistic features. Furthermore, even within the domain of language, it is challenging to identify cognitive measures that track disease severity across the PPA spectrum or even within syndromes (naming, for example, falls rapidly to floor in svPPA but this does not reflect daily life functionality).

We have recently created a symptom-led ‘PPA Progression Planning Aid’ (PPA-Squared), based on surveys of caregivers enrolled in PPA support groups in the United Kingdom and Australia about their lived experience of the illness [5, 112]. The proposed staging scheme and functional impairment severity scale cover three key domains of daily living (communication, nonverbal thinking and personality, and personal care and wellbeing) for each of the canonical PPA syndromes. For each syndrome, the scheme delineates six clinical stages covering all three functional domains; within each functional domain, we propose five severity levels (ranging from 0 (Presymptomatic) to 4 (Severe)) and associated functional milestones with implications for care (for example, all patients with later stage PPA are at risk of developing dysphagia; however, this occurs earlier in nfvPPA than other syndromes). This scheme will require prospective corroboration and validation in larger and more diverse patient cohorts, including atypical and mixed PPA cases.

Any staging scheme for PPA should be anchored in disease biology and objective biomarkers of disease evolution. Symptom-led stages should be correlated with neuropsychological scores, structural and functional neuroanatomy and extant rating scales for these diseases [109, 111, 113, 114], as well as CSF and other laboratory indices: the latter may include blood markers such as neurofilament light chain (NfL), which has shown some promise for disease monitoring though has not yet entered clinical practice [115]. Development of quantitative symptom severity scales would facilitate computational modelling approaches, which have been used successfully for tracking of progression in a number of neurodegenerative diseases [116, 117]. Multi-modality indices that integrate clinical measures with neuroimaging and other objective disease biomarkers may provide the most robust signals of disease progression and prognosis [118].

The factors that drive individual variability in the clinical expression and course of PPA are largely unknown, though constitutive genomic and epigenetic differences in language circuit organisation, interacting with specific vulnerabilities such as developmental dyslexia, may play a role [119]. One key question often posed by patients and families concerns life expectancy: this, too, varies widely from patient to patient; however, survival is modulated by language phenotype (on average, substantially longer in svPPA than other syndromes [4, 119, 120]), development of motor features [12] and atypical rightward cerebral lateralisation [121, 122] (probably associated with shorter survival). For reasons presently unclear, the tempo of illness progression seems to be particularly variable in lvPPA [123, 124].

### Beyond English

A major limitation of the literature on PPA to date has been its heavy emphasis on native English-speaking (and monolingual) patients. Epidemiological, clinical and neuropathological data on PPA in other languages are presently very incomplete. It is quite unlikely a priori that the clinical experience of English-speaking PPA can be simply or validly generalised to the wider world; rather, the linguistic characteristics and socio-cultural context of particular languages are anticipated to modify the presentation and course of the major syndromes. Indeed, the global spectrum of PPA might include syndromes that are rare or unrecognised in English-speaking patients. Clues to this are already evident: for example, nfvPPA in a Cantonese-speaking patient was associated with distinctive tone production, perception and dysgraphic deficits [125], and it has been suggested that dysgraphia phenotypes in Chinese language users might be used to screen for PPA and to classify PPA subtypes [126]. Word deafness and auditory agnosia appear to be relatively more common presentations of PPA in Japanese language users [84, 127–129]. On the other hand, phenomena such as ‘surface dyslexia/dysgraphia’ (the regularisation of irregularly spelled words when speaking or writing, due to breakdown of vocabulary knowledge, typically in svPPA) may take a different form in fully orthographically regular languages [130] and work is needed to identify equivalent cross-linguistic deficits. Encouragingly, published cohort studies devoted to PPA in languages other than English appear to be on the increase, both for European [12, 131–135] and other world languages [136–138]. In addition, screening instruments for PPA are being applied to other languages, both in translation [139] and as bespoke tools [140]. Looking forward, systematic international collaboration will be essential if we are to synthesise a comprehensive framework for PPA research relevant to diverse linguistic and cultural settings.

## Can brain pathology be predicted in PPA?

### Signatures of neuropathology

Predicting the underlying proteinopathy in PPA is potentially relevant to prognosis [119] and care planning, and will be increasingly required for the rational targeting of disease-modifying therapies. At present, however, this is far from straightforward.

Several phenotypic associations with neuropathology have been consistently observed across PPA series, albeit with varying estimates of their sensitivity and specificity [119, 141–144]; moreover, group-level associations potentially allow for considerable individual variation, so their value as ‘phenotypic biomarkers’ in clinical practice is uncertain [119]. The strongest and most consistent association links svPPA to TAR-DBA-binding-protein (TDP)-43 type C pathology, in line with the striking clinico-anatomical coherence of this syndrome [48, 49]; cases with predominant right-sided temporal lobe atrophy have more diverse neuropathological associations, supporting a distinct neurobiological status [38, 39, 145, 146]. lvPPA is widely regarded as a predictor of Alzheimer pathology and this association provides a neuropathological substrate for its extensive phenotypic overlap with typical amnestic and ‘visual’ variant Alzheimer syndromes [31, 32]. However, the strength of the association varies between series [8, 16]: an important minority of lvPPA cases have non-Alzheimer pathologies [30, 119, 147–149], while Alzheimer’s disease also presents as nfvPPA or ‘mixed’ PPA [141, 143]. In keeping with its clinical diversity, the pathology underpinning nfvPPA is heterogeneous: most patients will have a primary tauopathy such as progressive supranuclear palsy, corticobasal degeneration or (uncommonly) Pick’s disease, but a sizeable proportion of cases have TDP-43 or Alzheimer pathology [119, 141, 142]. These neuropathological associations of nfvPPA may partition according to the sub-syndromic variant phenotype: PPAOS is strongly associated with underlying tauopathy [19, 56], whereas progressive agrammatic aphasia (without apraxia of speech) may be more likely, based on limited evidence, to have TDP-43 pathology [23, 149]. The development of associated neurological features may provide a strong signal of the causative brain pathology, progressive supranuclear palsy syndrome reliably predicting tauopathy and motor neuron features TDP-43 pathology [62, 142, 150].

Most cases of PPA are sporadic [151, 152]; however, a significant minority are genetically mediated, with autosomal dominant inheritance. The responsible pathogenic mutation usually resides in one of the three major genes causing frontotemporal dementia: progranulin (*GRN*) (the most frequent genetic cause), chromosome 9 opening reading frame 72 (*C9orf72*) or (rarely) microtubule-associated protein tau (*MAPT*) [151–153]. Among the three canonical PPA syndromes, nfvPPA is the most likely to be hereditary; causative mutations in *GRN, MAPT* and (particularly in association with motor neuron features) *C9orf72* have been identified [148, 150, 153, 154]. Cases of more or less typical lvPPA associated with *GRN* mutations have been identified in a number of series [119, 148, 149, 152, 155]. svPPA is rarely genetic but *MAPT* mutations appear more likely to cause this than other PPA syndromes; pathogenic mutations in *C9orf72* and other genes have also been reported [4, 141, 156, 157].

### Pursuing the proteinopathy

After a clinico-anatomical syndromic diagnosis of PPA is reached, further investigations to determine the underlying proteinopathy may be appropriate (see Fig. 2). At present, this is mainly to assess for Alzheimer biomarkers in CSF (elevated phospho-tau, total tau and tau:beta-amyloid_1–42_ ratio; reduced beta-amyloid42:40 ratio) or on radio-ligand positron emission tomography with Pittsburgh compound B or fluorine 18-labelled tracers (florbetapir, florbetaben, and flutemetamol) (increased uptake with binding of beta-amyloid plaques), as this may help guide a trial of symptomatic therapy for Alzheimer’s disease. Genetic screening should be considered in younger patients, particularly where there is a suggestive family history of younger onset dementia in a first-degree relative (especially frontotemporal dementia or motor neuron disease), the PPA phenotype is atypical (as a genetic basis may be somewhat more likely in this scenario [148, 149, 153, 158, 159]) and/or there is strikingly asymmetric fronto-temporo-parietal atrophy on MRI (as this may signal a *GRN* mutation [142, 160]). However, the decision whether to broach genetic testing should always be weighed carefully: this is chiefly of clinical relevance for potentially at-risk family members (especially children). It should also be kept in mind that even where Alzheimer biomarkers are positive, conjoint (sporadic or genetic) pathologies may also be present and may drive the phenotype [30, 119, 143].

On balance, the value of both clinical characterisation and biomarker studies in predicting brain pathology across the PPA spectrum remains limited. Given the dearth of clear phenotypic predictors of neuropathology, and the issue of mixed pathologies [161], reliable and practical imaging and/or fluid biomarkers of non-Alzheimer tau and TDP-43 proteinopathies are sorely needed, particularly with a view to trials of disease-modifying therapies in PPA and other frontotemporal dementias. To date, however, these have remained elusive [162, 163].

## What is the core pathophysiology of PPA?

From a clinical perspective, a quest for fundamental neural mechanisms driving PPA syndromes may seem rather theoretical. We would argue, however, that clinicians have a stake in this enterprise—on account of the very complexity of PPA and the difficulties that surround diagnosis and neuropathological correlation. There are several motivations to move beyond traditional neurolinguistic accounts of these syndromes to address core pathophysiological mechanisms of PPA [164]. Neurolinguistic functions conventionally sampled in syndrome characterisation such as naming, grammar or speech repetition encompass a number of component processes. A pathophysiological account might dissect these and also suggest links between language dysfunction and the apparently disparate, non-linguistic cognitive and behavioural impairments that together constitute PPA syndromes. Further, by addressing the roots of these syndromes in neural circuit dysfunction, such an account might move closer to the underlying proteinopathies that target these circuits, open up fresh avenues for characterising PPA using physiologically informed methods (such as autonomic recordings, animal models, artificial neural networks, and functional neuroimaging [71, 75, 165–170]) and inform the identification of new physiological biomarkers. These (in contrast to conventional biomarkers) would provide a read-out of neuronal function, sufficiently dynamic to detect very early disease and to assess the impact of therapies, including novel indices such as perceptual learning of degraded speech (an index of retained cerebral plasticity [67]).

The neural networks implicated in the major PPA syndromes have been delineated in some detail, and network changes in these syndromes extend widely beyond the ‘hubs’ of maximal atrophy seen on clinical brain MRI scans [171, 172]. Each of the canonical PPA phenotypes is predominantly associated with a particular neural network and underlying proteinopathy (svPPA with the anterior temporal semantic appraisal network and TDP-43 type C; PPAOS with the dorsal prefrontal motor network and tauopathy; lvPPA with the temporo-parietal ‘default mode’ network and Alzheimer’s disease). This implies that the neural circuitry mediating the phenotype is preferentially vulnerable to that proteinopathy: a concept relevant to a broad range of neurodegenerative diseases, which we have termed ‘molecular nexopathy’ [172, 173]. Why and how pathogenic protein properties map onto neural circuit characteristics to create a molecular nexopathy is still poorly understood; however, this formulation has several important implications relevant to our understanding of PPA.

First, the neural circuits targeted in these PPA ‘nexopathies’ are engaged in a number of other cognitive operations besides language. Language functions depend on iterative transformations of sensory signals to meaning and/or actions, characterised neurophysiologically by spatio-temporal integration, nonlinear coding, plasticity and reciprocal interactions between processing stages [164, 174]; thus, language is likely a priori to be exquisitely vulnerable to the effects of neurodegenerative pathologies that disrupt neural circuit integrity. However, the physiological ‘lesion’ is likely to disrupt a number of additional, more generic cognitive and behavioural functions, potentially explaining why neurodegenerative pathologies that target this circuitry are heralded by speech and language deficits as part of a matrix of associated impairments.

Second, the speech and language deficits caused by proteinopathies need not closely resemble post-stroke aphasic syndromes (which, among other factors, are dictated by vascular anatomy and represent a sudden interruption rather than insidious erosion of neural network function [91]). Thus, we should anticipate that PPA syndromes will differ from the neurolinguistic syndromes of classical aphasiology. Relatedly, proteinopathies may target particular neural elements or connection types, rather than brain regions per se: PPA syndromes might, therefore, encompass deficits arising from different functional brain networks, potentially accounting for ‘atypical’ phenotypes (such as the aphasic syndrome associated with *GRN* mutations [15, 103, 159]). Vulnerability to proteinopathies may be modulated by neurodevelopmental vulnerabilities and genomic profiles, though any relationship is likely to be complex [119, 175–179].

We have previously proposed candidate core pathophysiological processes that might underpin each of the canonical PPA syndromes [164] (Fig. 3). These rest on a very general model of brain (neural circuit) operation, ‘predictive coding’, according to which stored predictions about incoming sensory data are compared iteratively with actual incoming data and updated so as to minimise error [180]. Perception as well as complex output behaviours such as speech can be understood using a predictive coding framework [169, 181]. In the case of the brain circuitry mediating language, we conceptualise predictive coding as a series of neural ‘template-matching’ operations, whereby incoming data are reconciled with stored representations (for phonemes, objects, motor routines, etc.) to generate an output. How neurodegenerative proteinopathies might degrade neural circuit operation to disrupt representations and thus ultimately language output and other behaviours has not been worked out in detail for PPA. In the case of svPPA—arguably the best defined of the canonical PPA syndromes clinically and pathologically—there is evidence to suggest how a complex disease phenotype with puzzling neuroanatomical focality [44] might be deconstructed to its pathophysiological building blocks, at the level of histopathological effects on neural circuit function [71, 166]. The semantic appraisal network—anchored in the anterior temporal lobes—depends for its operation on strongly convergent integration of sensory data regularities into multimodal semantic representations [49, 182]; such integration is supported by short range interneuronal inhibitory (GABA-ergic) connections that ‘channel’ neural information flow [183]. These connections are targeted by TDP-43 type C in svPPA [166], leading to an erosion of semantic representations that predicts the cognitive and behavioural manifestations of svPPA.

## How can PPA best be treated?

Our last question may surprise many neurologists. In fact, despite the present lack of effective disease-modifying therapies for PPA, there is much that can be done to help these patients live as well as possible with their illness. Management begins with the diagnostic process, building up a picture of the impact of the illness on this patient and their family, their social and occupational functioning, the profile of impairments and retained competencies. Once made, the diagnosis and its implications must be carefully and unhurriedly discussed, the general practitioner and appropriate local services and supports should be engaged and future planning should begin. We find it useful to review disease impacts under the broad categories of communication, nonverbal cognition, social and emotional behaviour and physical neurological features [5]: these require periodic review to address dynamically changing needs, as the illness unfolds. Psychological as well practical support should be offered to patients and caregivers, and many will benefit from contact with a local or national support group [184, 185]. A problem-based approach to symptoms might to some extent be applied in any patient with PPA [31, 45], though we find that the major syndromes have specific needs and capacities that tend to dictate management [5].

### Non-pharmacological strategies

Speech and language therapy has an integral part to play in the management of PPA [186], though there remain significant inequities and barriers to accessing it (not least a lack of knowledge about its potential benefits in PPA [187–189]). The speech and language therapist can assist with the diagnostic assessment [92], which will also facilitate the design of tailored interventions to support communication. Rather than one-size-fits-all, deficit-focussed or rehabilitative cognitive ‘re-training’ approaches developed for post-stroke aphasia, personalised ‘functional communication interventions’ that promote participation in activities and life situations by engaging a communication partner (usually, the patient’s primary caregiver) are more likely to be effective in PPA [186, 190–192]. Script training has been shown to improve speech fluency and grammar in nfvPPA [193] while interventions focussed on naming and word retrieval can improve communication in all PPA syndromes, particularly svPPA and lvPPA [194, 195]. Such interventions are most beneficial when introduced early in the illness, when they are customised to be personally relevant to the patient and if they can be scaled to the level of natural discourse [196–198]. Communication partner training such as Better Conversations with PPA addresses the essential role of those close to the patient in everyday social interactions: such training can identify communication barriers and promote strategies to overcome them across the PPA spectrum [189, 199–201], while addressing a key everyday management need identified by people with PPA and their care partners [202]. Therapists can also provide guidance in the use of ancillary communication devices, which may be a communication lifeline where the patient’s chief limitation is motor speech production (such as PPAOS [57]) Equally, however, ‘low tech’ communication aids should not be overlooked: a card, booklet or bracelet that the patient can carry may be crucial in maintaining independence. Interventions should, of course, be dynamically customised for evolving needs at different stages of PPA [5]. Later in the course of PPA, the speech and language therapist brings expertise in the management of dysphagia: anticipating problems (particularly in nfvPPA), assessing swallowing safety, and advising on appropriate eating and dietary strategies and assisted nutrition, including percutaneous endoscopic gastrostomy.

There is increasing interest in non-invasive neuromodulation with transcranial direct current or magnetic brain stimulation to improve language function in PPA, with reports of benefit in case studies and small cohorts [203–205]: coupled with behavioural interventions, such approaches might constitute a practical application of pathophysiological principles; however, larger trials are awaited. Similar considerations apply to the development of ‘smart’ assistive hearing devices and interventions to address central auditory dysfunction in PPA [206, 207].

Non-pharmacological strategies for other management issues that commonly arise in people with PPA, such as challenging behaviours, parkinsonism and mood disturbance, are similar to the wider frontotemporal dementia spectrum [208].

### Pharmacological treatment

On grounds of clinical pragmatism, we tend to offer a trial of symptomatic therapy with acetylcholinesterase inhibitors and/or memantine in patients with lvPPA and nfvPPA (where Alzheimer pathology is a consideration), although any benefit is usually modest and care is needed to avoid exacerbating concomitant behavioural or extrapyramidal features with acetylcholinesterase inhibitors [209, 210]. Behavioural and psychological symptoms such as depression, anxiety, impulsivity and aggression may be treated with serotonergic antidepressants or new-generation atypical antipsychotics, in parallel with non-pharmacologic approaches [211, 212]. Particular care is needed in the use of neuroleptic medications, as patients with PPA tend to be sensitive to extrapyramidal side effects.

The recent exciting developments towards effective disease modification based on phase III clinical trials of anti-amyloid monoclonal antibody therapy with lecanemab and donanemab in Alzheimer’s disease are a signal of hope for people living with PPA [85]. Patients with atypical Alzheimer variant syndromes (including lvPPA) may be eligible for these new disease-modifying treatments, though current guidelines also emphasise that safety and efficacy in these syndromes have not been specifically studied [213]. This also highlights the importance of early, expert diagnosis to determine whether an individual presenting with speech or language symptoms has a clinico-radiological phenotype consistent with primary Alzheimer pathology—with a view both to obtaining biomarker support and (if positive) determining whether this might be incidental to a concomitant culprit proteinopathy [30, 143]. There has been relatively less progress in disease-modifying therapy for non-Alzheimer pathologies in the frontotemporal dementia spectrum, but this is an increasingly active area of research, with a number of candidate agents in early stage trials targeting sporadic tau and TDP-43 pathologies as well as the pathogenic pathways of *GRN* and *C9orf72* mutations [214]. As well as specific protein biomarkers, bespoke staging and outcome measures appropriate for tracking change in PPA are urgently needed to support clinical trials [5]. Evidence for retained neural plasticity across the PPA spectrum [67, 167, 215] bodes well for the eventual deployment of disease-modifying agents in these diseases, potentially coupled with neurorehabilitation interventions to harness brain repair mechanisms.

### The next 40 years

The future of PPA research and clinical practice and ultimately, effective disease modification for people living with PPA will depend on finding answers to these and other questions. A reappraisal of these diseases seems in order—existing diagnostic criteria do not capture their complexity and heterogeneity, and this issue is practically relevant both for counselling of patients and caregivers and for the development of new diagnostic tools and markers. These are highly dynamic and often protean disorders—in the deficits they manifest, their time course, their sensitivity to developmental and contextual factors acting on the language system, and their care needs and response to interventions. The determinants of individual variation are still largely unknown but will likely be crucial in the design of personalised therapy. PPA is rare, meaning that no single centre can build up a comprehensive picture of its full clinical diversity and the common neurobiological threads that may bind syndromes—the field stands in urgent need of international, multi-centre collaboration, across the barriers of language, culture and socioeconomic status. We need systematic assessment protocols that encompass non-linguistic dimensions of nonverbal auditory communication, behaviour and physical disability; these often determine how well someone lives with the illness. We also need a much clearer picture of how illness evolves in the individual person—so that needs can be anticipated and supports engaged proactively, and to detect earliest stage disease reliably when opportunities for effective intervention are maximal. Staging in turn will support larger scale clinical trials in PPA. However, the promise of disease-modifying therapies targeting specific proteinopathies will only be realised with more detailed neurobiological models of PPA and how molecular pathologies link to phenotypes—in this enterprise, genetic forms of PPA might serve as instructive test cases, and mixed phenotypes warrant more attention, as these often signal underlying Alzheimer pathology [141, 143]. Designing rational interventions for PPA—both pharmacological and non-pharmacological—will entail an appreciation of preserved capacities as well as impairments [57]. This speaks to the wider theme of pathophysiology, which we would argue underwrites all of the questions posed here and offers the best prospect of a transformative solution—both by providing rapid and specific readouts of neural dysfunction to guide therapeutic decision making, and by informing the search for novel intervention targets and brain repair and compensatory mechanisms [67, 216]. Clinical observation will remain essential, not least in revealing fresh facets of these kaleidoscopic diseases [217]. There are grounds for optimism that the next 40 years will see the ‘language-led dementias’ rendered treatable.

### Supplementary Information

Below is the link to the electronic supplementary material.

Supplementary file1 (DOCX 373 KB)

## Data Availability

Not applicable.
