# Supplementary material for: Primary progressive aphasia: six questions in search of an answer
Source: J Neurol. 2023 Oct 31;271(2):1028–46. doi: 10.1007/s00415-023-12030-4 (PMC10827918; doi:10.1007/s00415-023-12030-4)
Supplement: Supplementary file 1 — Supplementary file1 (DOCX 373 KB) [file 415_2023_12030_MOESM1_ESM.docx]

**Supplementary Material**

**Primary progressive aphasia: six questions in search of an answer,**

**by CRS Belder et al**

**Table S1.** Diagnosis of primary progressive aphasia based on current consensus criteria

| Gateway diagnosis | **Primary progressive aphasia** | | |
| --- | --- | --- | --- |
| **Inclusion**  *All of:* | Most prominent clinical feature is difficulty with language | | |
|  | This is the principal cause of impaired daily living activities | | |
|  | Aphasia was the most prominent deficit at symptom onset and for initial phases of illness | | |
| **Exclusion**  *None of:* | Pattern of deficits better accounted for by another, nondegenerative disorder | | |
|  | Cognitive disturbance better accounted for by a psychiatric diagnosis | | |
|  | Prominent initial episodic memory, visual memory, visuoperceptual impairments | | |
|  | Prominent initial behavioural disturbance | | |
| Syndromic diagnosis | **Nonfluent variant** | **Semantic variant** | **Logopenic variant** |
| **Clinical** | *At least one of:* | *Both of:* | *Both of:* |
| *Core features* | Agrammatism in language production | Impaired confrontation naming | Impaired single-word retrieval in spontaneous speech and naming |
|  | Effortful, halting speech with inconsistent speech sound errors and distortions (speech apraxia) | Impaired single-word comprehension | Impaired repetition of sentences and phrases |
| *Other features* | *At least two of:* | *At least three of:* | *At least three of:* |
|  | Impaired comprehension of syntactically complex sentences | Impaired object knowledge, particularly for low-frequency or low-familiarity items | Speech (phonologic) errors in spontaneous speech and naming |
|  | Spared single-word comprehension | Surface dyslexia or dysgraphia | Spared single-word comprehension and object knowledge |
|  | Spared object knowledge | Spared repetition | Spared motor speech |
|  |  | Spared speech production (grammar and motor speech) | Absence of frank agrammatism |
| **Imaging-supported** | *At least one of:* | *At least one of:* | *At least one of:* |
|  | Predominant left posterior fronto-insular atrophy on MRI | Predominant anterior temporal lobe atrophy | Predominant left posterior peri-sylvian or parietal atrophy on MRI |
|  | Predominant left posterior fronto-insular hypoperfusion/metabolism on SPECT /PET | Predominant anterior temporal hypoperfusion/metabolism on SPECT /PET | Predominant left posterior peri-sylvian or parietal hypoperfusion/ metabolism on SPECT/PET |
| **Pathologically definite** | *At least one of:* | *At least one of:* | *At least one of:* |
|  | Histological evidence of specific neurodegenerative pathology | Histological evidence of specific neurodegenerative pathology | Histological evidence of specific neurodegenerative pathology |
|  | Known pathogenic mutation | Known pathogenic mutation | Known pathogenic mutation |

The Table summarises current consensus diagnostic criteria (Gorno-Tempini et al. 2011) for the major syndromes of primary progressive aphasia, based on clinical, neuroimaging and neuropathological features. The criteria allow for different levels of diagnosis of primary progressive aphasia: an initial ‘gateway’ which must be passed in order to diagnose any form of primary progressive aphasia, and syndromic classification based on the pattern of clinical language deficits in each of the major variants, which may be supported by brain imaging and/or histopathological findings.
